# Relation of the kynurenine pathway with cognitive functioning and differences in patients with cognitive impairment or dementia: a systematic review and meta‐analysis

**DOI:** 10.1002/alz70856_102238

**Published:** 2025-12-25

**Authors:** Lieke Bakker, Kyonghwan Choe, Daniel L.A. van den Hove, Simone J.P.M. Eussen, Gunter Kenis, Inez H.G.B. Ramakers, Frans R.J. Verhey, Bart P.F. Rutten, Sebastian Köhler

**Affiliations:** ^1^ Alzheimer Center Limburg, Maastricht University, Maastricht, Limburg, Netherlands; ^2^ Mental Health and Neuroscience Research Institute (MHeNs) and European Graduate School of Neuroscience (EURON), Faculty of Health, Medicine and Life Sciences (FHML), Maastricht University, Maastricht, Limburg, Netherlands; ^3^ Cardiovascular Research Institute Maastricht (CARIM) and Care and Public Health Research Institute (CAPHRI), Maastricht University, Maastricht, Limburg, Netherlands

## Abstract

**Background:**

The kynurenine pathway (KP) is associated with pathophysiological processes underlying dementia, but results from clinical studies are contradictory. Therefore, this systematic review and meta‐analysis summarized the available evidence for differences in KP metabolites in patients with cognitive impairment compared to cognitively healthy individuals and associations between KP metabolites and cognitive functioning.

**Method:**

Studies published in English with prospective, cross‐sectional, or case‐control designs that were published in Pubmed, Embase, PsychINFO, or the Cochrane Database of Systematic Reviews up to October 2023, were included. Random‐effects meta‐analyses of standardized mean differences (SMD) were done. Heterogeneity, meta‐regression, small study bias, and study quality assessments were carried out.

**Result:**

Of 8797 studies, 98 met inclusion criteria. Meta‐analyses comparing Alzheimer's disease (AD) dementia patients to controls (*n* = 27 studies) indicated lower cerebrospinal fluid (CSF) levels of tryptophan (SMD = ‐0.26 [95% CI ‐0.41, ‐0.12]), 3‐hydroxykynurenine (‐0.21 [‐0.39, ‐0.04]), anthranilic acid (‐0.28 [‐0.48, ‐0.08]), and quinolinic acid (‐0.38 [‐0.56, ‐0.21]) in AD dementia, while CSF levels of kynurenic acid were higher (0.18 [0.01, 0.35]). Blood levels of tryptophan (‐0.39 [‐0.51, ‐0.28]), kynurenic acid (‐0.31 [‐0.47, ‐0.15]), xanthurenic acid (‐0.34 [‐0.54, ‐0.15]), and 3‐hydroxyanthranilic acid (‐0.42 [‐0.61, ‐0.22]) were lower in AD dementia. For some of these metabolites, similar directions were observed in meta‐analyses comparing individuals with mild cognitive impairment with controls, although the number of included studies in these analyses was relatively small (*n* = 11). Associations with cognitive test scores were inconclusive and generally non‐significant.

**Conclusion:**

These results suggest that AD dementia is associated with lower blood levels of several KP metabolites. Findings also challenge assumptions that neurotoxic quinolinic acid levels are an important contributor to dementia pathogenesis.

